# Editorial: Lipid Metabolism in Development and Environmental Stress Tolerance for Engineering Agronomic Traits

**DOI:** 10.3389/fpls.2021.739786

**Published:** 2021-08-24

**Authors:** Zhi-Yan Du, Susanne Hoffmann-Benning, Shiwen Wang, Lina Yin, Agnieszka Zienkiewicz, Krzysztof Zienkiewicz

**Affiliations:** ^1^Department of Molecular Biosciences and Bioengineering, University of Hawaii at Manoa, Honolulu, HI, United States; ^2^Department of Biochemistry and Molecular Biology, Michigan State University, East Lansing, MI, United States; ^3^Institute of Soil and Water Conservation, Northwest A&F University, Yangling, China; ^4^Centre for Modern Interdisciplinary Technologies, Nicolaus Copernicus University in Toruń, Toruń, Poland

**Keywords:** lipid biosynthesis and turnover, membrane and storage lipids, development, biotic and abiotic stress, genetic engineering

Lipids are the essential building blocks of cellular membranes, and they play fundamental roles in numerous biological activities such as photosynthesis, protection, environmental and cellular communication, and storage of carbon and energy. Lipid metabolism is a dynamic and complicated process that includes lipid biosynthesis, transport, accumulation, turnover, and excretion, acting to regulate plant development and tolerance to various environmental stresses. Understanding how lipid metabolism regulates the development and growth in response to adverse conditions raises many interesting questions. Communication between membrane and storage lipids has been observed during the progress, for example, membrane degradation accompanied by lipid droplet (LD) accumulation. Recently, autophagy has been connected with lipid metabolism in plants and algae under stress conditions (Zienkiewicz and Zienkiewicz). Because of the vital role of lipid metabolism in photosynthetic organisms, exploring lipid processes is very important for engineering crops to obtain better agronomic traits including productivity, nutrition, stress tolerance and resilience.

This Research Topic includes 10 original review and research articles, with a special focus on lipid metabolism in crops. The review by Zienkiewicz and Zienkiewicz summarizes the most recent advances on LD degradation, which is a key process for the release of triacylglycerol (TAG). As the major reservoir of cellular carbon and energy, TAG is packed in LD, a dynamic organelle at the center of cellular metabolism. The review also compares the LD degradation in plants and algae (Figure 1A). Looking at lipid metabolism in the context of membrane composition and transport, Salvaing et al. report the function of the Arabidopsis ALA10, a phospholipid flippase of the P_4_ type-ATPase family, in reducing the 18:2 desaturation of phosphatidylcholine in the endoplasmic reticulum (ER), while Liao et al. show the roles of two rice acyl-CoA-binding proteins, OsACBP4 (plasma membrane and ER) and OsACBP5 (apoplast), in the intra- and extracellular transport of acyl-CoA esters, respectively (Figure 1B). Other contributions directly address the role of lipid metabolism during development, *EgMIXTA1*, an R2R3 MYB-type transcription factor, promotes the cuticular wax formation in *Eustoma grandiflorum* (lisianthus) leaves (Wang et al.); together with the specificities of extraplastidial acyltransferase enzymes, the olive *OeFAD2-2* and *OeFAD2-5* regulate the oleic/linoleic acid ratio in the mesocarp of olive cultivars (Figure 1C) (Hernández et al.). The Research Topic also highlights the significance of lipid metabolism in environmental stress response and tolerance (Figure 1D). In *Arabidopsis*, expression of *CsKCS6*, a gene coding the 3-Ketoacyl-CoA synthase (KCS) that catalyzes the biosynthesis of very-long-chain fatty acid (VLCFA) in *Citrus sinensis* (Newhall navel orange), reduces water loss and ion leakage and increases tolerance to drought and salt stress (Guo et al.); *Arabidopsis* phospholipid:diacylglycerol acyltransferase 1 (AtPDAT1) promotes the activities of acyl-CoA:lysophosphatidylcholine acyltransferase (LPCAT) and acylCoA:lysophosphatidylethanolamine acyltransferase (LPEAT) and improves resilience to heat and cold exposure (Demski et al.). In *Vaccinium corymbosum* L., cv. Duke (Duke blueberry), intermittent warming treatment of the fruit regulates its lipid metabolism such as increases in phosphatidylcholine, linoleic acid, and oleic acid, which enhances the low-temperature tolerance in the fruit (Dai et al.). In the roots of maize, integrated lipidomic and transcriptomic analyses reveal the membrane lipid remodeling and gene regulations under saline-alkaline (Xu et al.) or cold stress (Zhao et al.), which are applicable for engineering the crop for better performance under these stress conditions.

**Figure 1:**
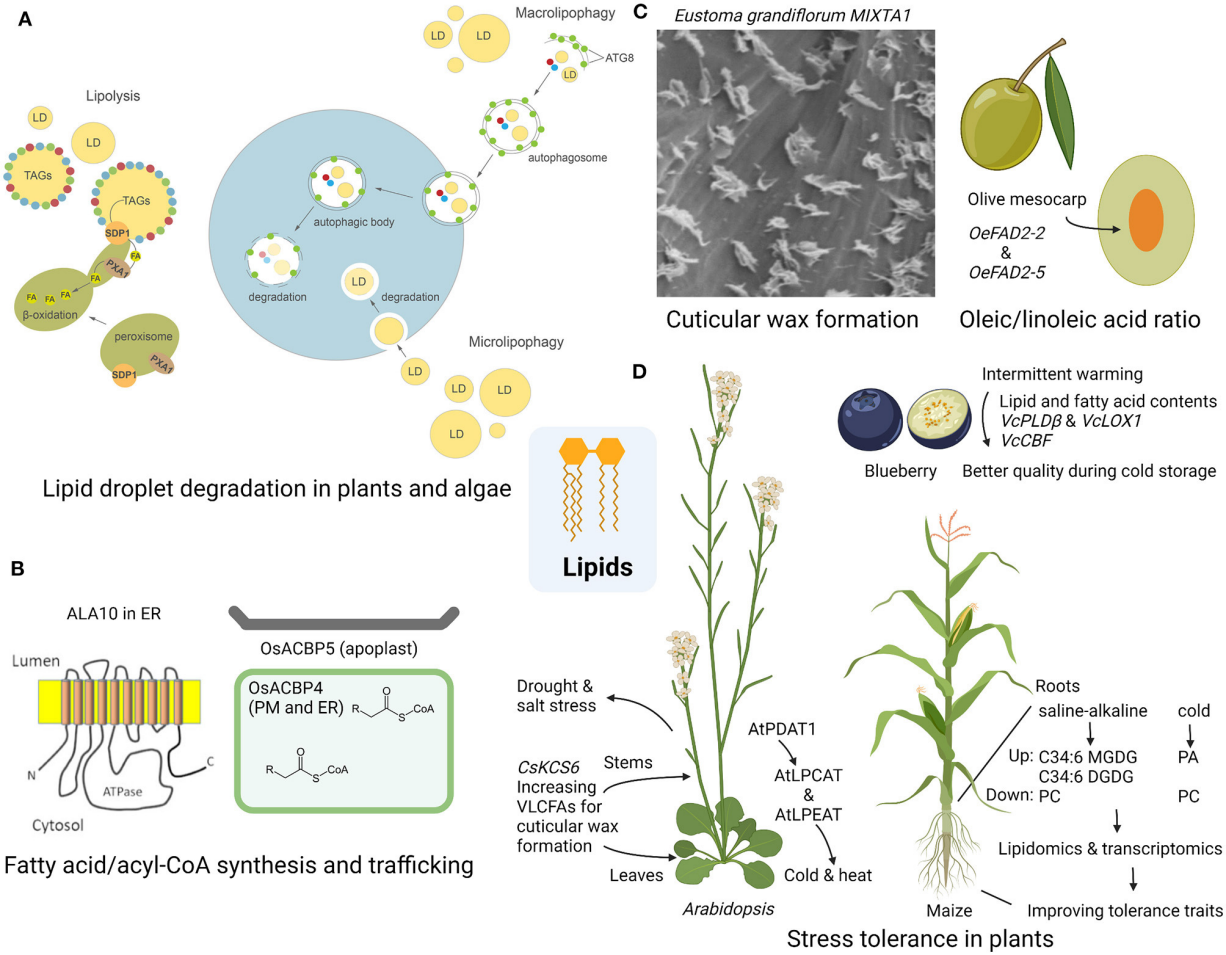
Overview of the articles in this Research Topic. **(A)** Lipid droplet degradation in plants and algae (Zienkiewicz and Zienkiewicz). **(B)** Biosynthesis and trafficking of fatty acids (Salvaing et al.) and acyl-CoAs (Liao et al.). **(C)** Lipid metabolism in cuticular wax formation in *Eustoma grandiflorum* (Wang et al.) and fatty acid composition in olive mesocarps (Hernández et al.). **(D)** Understanding the lipid and fatty acid dynamics in different plants including *Arabidopsis* under drought-salt (Guo et al.) and cold stress (Demski et al.), blueberry fruit during cold storage (Dai et al.), and maize under saline-alkaline (Xu et al.) or cold stress (Zhao et al.).

The collection of articles in this Research Topic demonstrates the significance of lipid metabolism in various biological processes and the findings will contribute to engineering agronomic traits of crops for the increasing demand for food and bioproducts.

## Author Contributions

All authors listed have made a substantial, direct and intellectual contribution to the work, and approved it for publication.

## Conflict of Interest

The authors declare that the research was conducted in the absence of any commercial or financial relationships that could be construed as a potential conflict of interest.

## Publisher's Note

All claims expressed in this article are solely those of the authors and do not necessarily represent those of their affiliated organizations, or those of the publisher, the editors and the reviewers. Any product that may be evaluated in this article, or claim that may be made by its manufacturer, is not guaranteed or endorsed by the publisher.

